# Remarkable Presentation: Anaplastic Thyroid Carcinoma Arising from Chronic Hyperthyroidism

**DOI:** 10.1155/2018/7261264

**Published:** 2018-04-01

**Authors:** Habib G. Zalzal, Jeffson Chung, Jessica A. Perini

**Affiliations:** ^1^Department of Otolaryngology-Head and Neck Surgery, West Virginia University School of Medicine, Morgantown, WV, USA; ^2^Section of Endocrinology, Department of Internal Medicine, West Virginia University School of Medicine, Morgantown, WV, USA

## Abstract

**Background:**

Undifferentiated anaplastic carcinoma rarely develops from chronic hyperthyroidism. Although acute hyperthyroidism can develop prior to anaplastic transformation, chronic hyperthyroidism was thought to be a protective measure against thyroid malignancy.

**Methods:**

A 79-year-old female presented acutely to the hospital with dyspnea. She had been taking methimazole for chronic hyperthyroidism due to toxic thyroid nodules, previously biopsied as benign. Upon admission, imaging showed tracheal compression, requiring a total thyroidectomy with tracheostomy for airway management.

**Results:**

Pathology demonstrated undifferentiated anaplastic thyroid carcinoma. The patient passed away shortly after hospital discharge. Despite treatment with methimazole for many years, abrupt enlargement of her toxic multinodular goiter was consistent with malignant transformation. Chronic hyperthyroidism and toxic nodules are rarely associated with thyroid malignancy, with only one previous report documenting association with anaplastic thyroid carcinoma.

**Conclusion:**

Progressive thyroid enlargement and acute worsening of previously controlled hyperthyroidism should promote concern for disease regardless of baseline thyroid function.

## 1. Introduction

Historically, chronic hyperthyroidism had been considered protective against thyroid carcinoma. Some data suggest a lower incidence of papillary thyroid cancer in those with lower TSH levels [1]. This presumably arises from a decreased stimulatory effect on thyroid tissue presented by the low serum thyroid stimulatory hormone (TSH) found in hyperthyroidism. However, with the increasing incidence of thyroid cancer seen over the past years, there appears to be an improved understanding that thyroid carcinomas can also arise in glands that are thyrotoxic due to Graves' disease, toxic multinodular goiter, and autonomously functioning thyroid adenomas [2]. Recent review of the literature has shown the risk of malignancy associated with toxic hot nodules ranges from 1 to 10.3% [2] or even up to 15% in those with Graves' disease [3]. Most of these documented malignancies are differentiated thyroid carcinomas and very rarely medullary thyroid cancer [4].

Anaplastic thyroid carcinoma (ATC) represents one of the most aggressive endocrine tumors and constitutes approximately between 1.6 and 5% of all thyroid malignancies [5]. ATC comes with a dismal prognosis limited to a 10–20% mean survival at 12 months [6]. Patients with ATC experience significant local compressive symptoms due to a rapidly evolving central neck mass (77%) along with dysphagia (40%), hoarseness (40%), and stridor (24%) [7]. Metastases are noted in 50% of patients at the time of diagnosis, most commonly in the lungs (80%), bone (6–16%), and brain (5–13%) [7]. Not uncommonly patients also develop thyrotoxicosis. Although thyrotoxicosis can develop in a gland with ATC, it is rare to find ATC develop from underlying longstanding hyperthyroidism. We found only one other case in the literature of ATC arising from a patient with chronic hyperthyroidism [8]. In this report, we present our experience with a patient who developed ATC after many years of hyperthyroidism and toxic multinodular goiter.

## 2. Patient

A 79-year-old Caucasian female presented to our institution with chest pain, dyspnea, and a rapidly enlarging thyroid goiter. The patient endorsed that she was previously quite healthy aside from stable hyperthyroidism and a nonenlarging thyroid goiter. Her thyroid had been overactive for years, treated with stable dose of 7.5 mg daily of methimazole for at least the past four years. In 2002, 2003, and 2010, she had had thyroid uptake scans indicating heterogeneous elevated uptake consistent with toxic multinodular goiter and a cold area was identified in the lower right lobe. In 2000 and 2010, she had biopsies of the nodules of the right and left lobes with benign results. Since treatment with methimazole, she had been asymptomatic up until three months prior to presentation, living a mostly independent and having active lifestyle that included cooking, gardening, and daily errands.

The patient's family became concerned approximately three months before presentation to our facility because the patient lost a significant amount of weight while her goiter rapidly enlarged. Soon after, she had sudden “terrible pain” in the left side of her neck, diagnosed as an internal jugular vein thrombus. At the time of this diagnosis, her thyroid measured 8.4 × 12 cm. She saw her endocrinologist who noted that her thyroid was hyperfunctioning again and no longer controlled with her daily dose of methimazole, which was subsequently increased. A month later, her labs showed that her thyroid was even more overactive. Her dose of methimazole was subsequently increased to 30 mg per day. She was evaluated for surgery the following month, but surgery was deferred due to uncontrolled hyperthyroidism. The day prior to presentation at our institution she developed chest pressure and tightness when lying down. A computed tomography (CT) scan of her chest and neck showed that her thyroid had grown to 9.8 × 13.2 cm.

**Figure 1:**
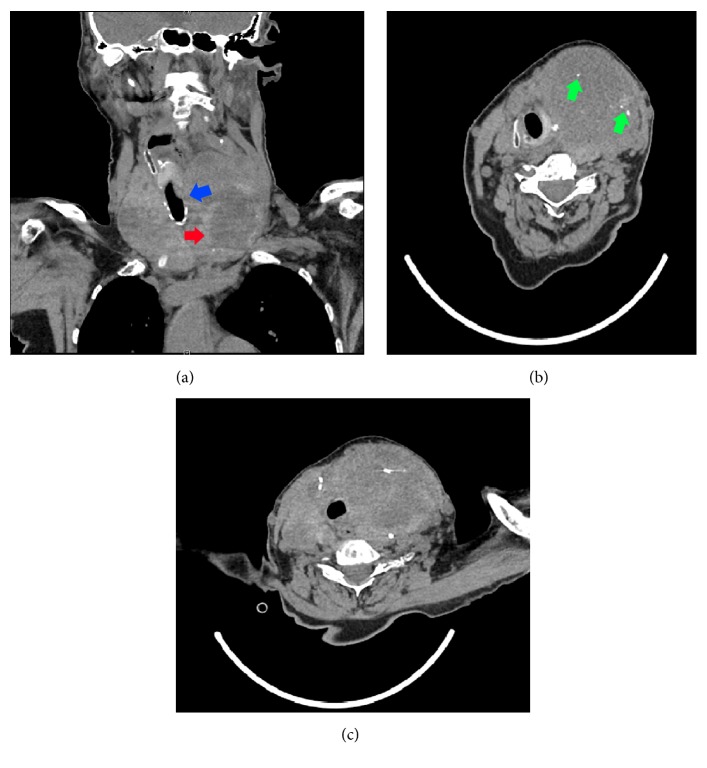
Significantly enlarged thyroid mass causing tracheal deviation to the right (blue arrow) with hypoattenuation within the left thyroid lobe (red arrow) (a). Scattered calcifications (green arrows) apparent within the lesion (b) with extension into the anterior mediastinum and continued deviation of the trachea (c).

Upon admission, her TSH measured <0.003 *μ*IU/L (reference range 0.5–4.70 *μ*IU/mL), free serum thyroxine (T_4_) measured 1.56 ng/dL (reference range 0.7–1.25 ng/dL), and free serum triiodothyronine (T_3_) measured 2.4 pg/mL (reference range 1.7–3.7 pg/mL). Ultrasound of her thyroid showed gross enlargement of both thyroid lobes with the largest nodules measuring 9.7 cm in the left lobe and 5.1 cm in the right lobe. Subsequent CT scan of her chest showed the mass extending into the anterior mediastinum with scattered calcifications within the mass (Figure 1). There was narrowing of the trachea in addition to multiple pulmonary masses throughout the visualized lung fields. She was continued on methimazole (20 mg twice a day) while hospitalized and started on propranolol and prednisone for symptomatic control. Her free T_4_ improved and free T_3_ remained within normal limits on this regimen.

**Figure 2:**
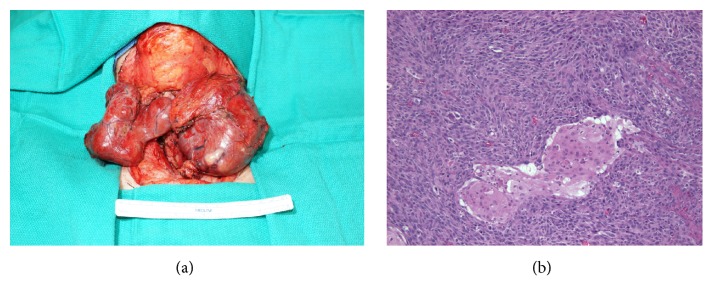
(a) Intraoperative photograph of total thyroid specimen, greatest dimension 8.7 cm in size. (b) Microscopic pathology of squamoid and undifferentiated thyroid cells.

**Figure 3:**
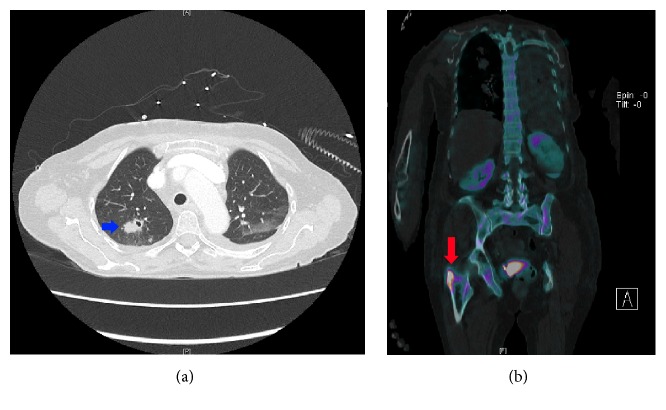
Metastatic work-up demonstrates a pulmonary nodule within the right upper lobe (1.1 × 2.1 cm) with central cavitation (blue arrow) (a) and increased 99 mTc HDP uptake within the right intratrochanteric femur positive for a destructive lytic lesion (red arrow) (b).

Due to dyspnea and malignancy concerns, she underwent a total thyroidectomy two weeks after admission to obtain a pathologic diagnosis. Intraoperative findings included involvement of the left recurrent laryngeal nerve and tracheal invasion. Intraoperative biopsies sent for frozen section analysis were reported as poorly differentiated carcinoma, suggestive of anaplastic thyroid carcinoma (Figure 2). As a result, a prophylactic tracheostomy was placed. The final pathology report confirmed undifferentiated anaplastic carcinoma (8.7 cm) involving the left thyroid, positive for lymphatic involvement (2/5 central nodes) and extrathyroidal extension to the pretracheal cartilage. Surgical margins were positive along the left middle and inferior pole. The right thyroid lobe had multiple benign nodes. Subsequent whole-body CT and nuclear medicine scan found multiple bilateral pulmonary nodules with central cavitation and a destructive right intertrochanteric lytic lesion with pathological fracture (Figure 3). The final staging was pT4b N1b M1 anaplastic thyroid carcinoma (American Joint Committee on Cancer 7th Edition). Chemotherapy was recommended for her ATC, as she was deemed not a candidate for radiation therapy. The patient's family chose to pursue chemotherapy closer to home. The patient quickly deteriorated towards the end of her hospitalization and missed several follow-up appointments with her medical oncologist upon discharge. She passed away at home within a month after leaving the hospital.

## 3. Discussion

Chronic hyperthyroidism had long been thought to be protective against the development of malignancy within the thyroid gland [2]. Thus, development of thyroid cancer within a hyperthyroid gland was thought to be uncommon. However, meta-analyses have shown that thyroid cancer can in fact develop in hyperthyroid glands, although it is not common, and most of the malignancies identified in hyperfunctioning thyroid glands have been differentiated thyroid cancers. Conversely, acute thyroiditis can develop in association with thyroid malignancies. For example, rare cases of “malignant pseudothyroiditis” have been described wherein local parathyroid malignancies or metastatic lesions to the thyroid prompted thyroid inflammation and thyrotoxicosis [9]. ATC is also associated with acute thyrotoxicosis that develops around the time of acute malignant transformation [2, 10]. A 2007 review of the association between ATC and thyrotoxicosis outlines eight cases in the literature [10]. The authors discuss the development of acute thyrotoxicosis with anaplastic thyroid cancer as a consequence of rapid leakage of thyroid hormone into the bloodstream from destroyed thyrocytes. Phillips et al. argue that the acute hyperthyroid state in ATC is caused by a hyperfunctioning metastatic tumor [10]. Thyrotoxicosis associated with ATC has been termed “anaplastic pseudothyroiditis” [11].

This situation, however, differs from our patient in that thyrotoxicosis did not originate from the anaplastic tumor but predated the malignant transformation by several years. Our case shows the unique condition in which anaplastic thyroid cancer developed from a hyperthyroid patient despite several years of methimazole treatment. ATC is associated with a previous history of thyroid goiter and is known to be more common in geographic regions of endemic iodine deficiency [7] but not associated with glands that have previously been hyperfunctioning.

To our knowledge, a 2014 report by Marcelino et al. is the only published case that associates chronic hyperthyroidism with subsequent development of ATC [8]. Their study reports a similar presentation in a 70-year-old male who had been diagnosed with a toxic nodule three years prior to diagnosis of ATC. He was conservatively managed with methimazole and *β*-adrenergic blockers. Three years later, he re-presented with dyspnea, hoarseness, and dysphagia similar to our patient. A subsequent neck CT scan showed a suspicious mass involving the left thyroid lobe and isthmus (7 × 6 × 5 cm), with invasion of the surrounding soft tissue, trachea, and recurrent nerve. Their patient was not a surgical candidate and underwent radiotherapy without much improvement in thyroid size before passing away several weeks later of airway compression [8]. Similar to Marcelino et al., previous work-up of our patient's thyroid nodules, including biopsies of bilateral nodules, was negative for malignancy. While her thyroid uptake scans previously identified a cold nodule in the lower right thyroid lobe, her surgical pathologic specimen of the left thyroid lobe identified the anaplastic carcinomic tissue while her right thyroid nodule was negative for anaplastic disease. In addition, previous thyroid scans had elevated and patchy uptake in the left lobe despite the pathologic finding. The development of ATC from differentiated thyroid carcinoma is well established [7], although our patient did not have this diagnosis, based on the negative FNA years prior. Whether our patient developed “de novo” ATC similar to what was theorized by Marcelino et al. is debatable, but plausible considering the fact that the previously toxic left thyroid nodule was the one which developed ATC in our patient. This was despite the presence of a cold nodule in the right thyroid lobe which remained negative for malignancy after removal.

Undifferentiated anaplastic thyroid carcinoma is a rare and underreported condition in the setting of chronic hyperthyroidism. There exists only one other reported case of this association in the literature, also involving a septuagenarian who developed rapid thyroid enlargement with tracheal compression years after diagnosis and control of hyperthyroidism with methimazole. While current recommendations for treatment of hyperthyroidism involve medical therapy, the presence of any nontoxic nodule in the thyroid, and any toxic nodule with suspicious features, should warrant work-up with ultrasound, fine needle aspirate, and ongoing surveillance. Our case demonstrates that even those with negative biopsy and longstanding hyperthyroidism are at risk of development of anaplastic thyroid carcinoma.
